# Unveiling the role of *Gardnerella vaginalis* in polymicrobial Bacterial Vaginosis biofilms: the impact of other vaginal pathogens living as neighbors

**DOI:** 10.1038/s41396-018-0337-0

**Published:** 2019-01-22

**Authors:** Joana Castro, Daniela Machado, Nuno Cerca

**Affiliations:** 1 Centre of Biological Engineering (CEB), Laboratory of Research in Biofilms Rosário Oliveira (LIBRO), University of Minho, Campus de Gualtar, 4710-057 Braga, Portugal; 2 Instituto de Ciências Biomédicas Abel Salazar (ICBAS), University of Porto, Rua de Jorge Viterbo Ferreira 228, 4050-313 Porto, Portugal

**Keywords:** Pathogenesis, Microbial ecology, Biofilms

## Abstract

Bacterial vaginosis (BV) is characterized by a highly structured polymicrobial biofilm, which is strongly adhered to the vaginal epithelium and primarily consists of the bacterium *Gardnerella vaginalis*. However, despite the presence of other BV-associated bacteria, little is known regarding the impact of other species on BV development. To gain insight into BV progress, we analyzed the ecological interactions between *G. vaginalis* and 15 BV-associated microorganisms using a dual-species biofilm model. Bacterial populations were quantified using a validated peptide nucleic acid fluorescence in situ hybridization approach. Furthermore, biofilm structure was analyzed by confocal laser scanning microscopy. In addition, bacterial coaggregation ability was determined as well as the expression of key virulence genes. Remarkably, our results revealed distinct biofilm structures between each bacterial consortium, leading to at least three unique dual-species biofilm morphotypes. Furthermore, our transcriptomic findings seem to indicate that *Enterococcus faecalis* and *Actinomyces neuii* had a higher impact on the enhancement of *G. vaginalis* virulence, while the other tested species had a lower or no impact on *G. vaginalis* virulence. This study casts a new light on how BV-associated species can modulate the virulence aspects of *G. vaginalis*, contributing to a better understanding of the development of BV-associated biofilms.

## Introduction

Bacterial vaginosis (BV) is the most common vaginal infection in women of reproductive age worldwide [1]. It is associated with adverse outcomes relating to both pregnancy [2] and fertility [3]. BV is characterized by a dramatic shift in the vaginal microbiota population, which involves the loss of beneficial bacteria (normally *Lactobacillus*-dominated) and a simultaneous proliferation of a complex mixture of other microorganisms [4–8]. The specific roles of the multiple microorganisms associated with BV, such as *Atopobium vaginae*, *Prevotella bivia, Mobiluncus mulieris, Corynebacterium amycolatum, Fusobacterium nucleatum, Dialister, Enterococcus faecalis, Eggerthella, Leptotrichia, Megasphaera*, and *Ureaplasma urealyticum*, are largely unknown [9–11] with *Gardnerella vaginalis* currently the best-studied species [5, 12–15]. The role of *G. vaginalis* in BV is not without controversy, however. First proposed as the sole etiological agent by Gardner and Dukes [16] and later studied by cultivation-independent approaches [17–19], its presence in healthy women cast doubt on its virulence potential [13, 20]. Nevertheless, in the past decade, it has been demonstrated that *G. vaginalis* had a significantly higher virulence potential than many other BV-associated species, as defined by higher initial adhesion and cytotoxic effect, as well as a greater propensity to form a biofilm [21–23]. Furthermore, recent genomic analysis revealed four *G. vaginalis* genome groups, with great differences between each other [19, 24–26]. Herein, we will refer to BV-associated *G. vaginalis* (BVGv) as isolates from women with BV that were previously phenotypically characterized as virulent isolates [13].

Due to its strong adherence to vaginal cells and biofilm-forming capacities, it has been suggested that BVGv initiates the colonization of the vaginal epithelium and serves as a scaffold to which other species subsequently can attach [11, 27–29]. As a result, during BV, there is a complex interplay between pathogenic species, endogenous vaginal microbiota, and the vaginal epithelium [5, 30]. Due to the presumably central role of BVGv in BV development, it is crucial to assess how secondary BV-associated microbial species interact with BVGv. The study of these microbial interactions is extremely important for obtaining knowledge of the pathogenicity of microbes in the host and for the development of effective treatments without relapses, a common problem in BV [31, 32]. Some studies have already evaluated the interplay between *G. vaginalis* and other BV-associated species in biofilms [33–36]. However, all these studies were carried out by observing a few phenotypic aspects of the interactions between *G. vaginalis* and BV-associated species, and, as such, more detailed studies are needed.

We recently showed that *G. vaginalis* exhibits a specific gene expression behavior according to its phenotype form, probably to overcome the host defenses and allow the colonization of mucosal tissue [37]. However, hardly any information exists on how BVGv gene expression is influenced by the presence of other BV-associated bacteria. It has been previously shown that co-culturing dual-species biofilms involves very specific alterations in gene expression, as observed in the oral biofilms microbiome [38], between staphylococcal species [39] and in *Veillonella parvula* and *Streptococcus mutans* consortia [40]. Therefore, we hypothesize that molecular interactions between *G. vaginalis* and multiple BV-associated bacteria could be very specific, highlighting possible key roles in BV development by some secondary anaerobes. Thus, in an effort to better understand the virulence of BVGv in polymicrobial communities, the aim of the present study was to analyze the interactions between *Actinomyces neuii, Atopobium vaginae, Brevibacterium ravenspurgense, Corynebacterium amycolatum, Corynebacterium tuscaniense, Enterococcus faecalis, Mobiluncus mulieris, Nosocomicoccus ampullae, Prevotella bivia, Propionibacterium acnes, Staphylococcus hominis, Staphylococcus saprohyticus, Staphylococcus simulans, Staphylococcus warnerii, Streptococcus anginosus,* and BVGv using a dual-species biofilm assembly.

## Methods

### Bacterial strains and culture conditions

*G. vaginalis* strain UM241 was isolated from a woman diagnosed with BV [13]. Fifteen more bacterial species associated with BV were included in this study, namely: *A. neuii, A. vaginae, B. ravenspurgense, C. amycolatum, C. tuscaniense, E. faecalis, N. ampullae, P. acnes, S. hominis, S. saprohyticus, S. simulans, S. warnerii, S. anginosus, M. mulieris*, and *P.bivia*. More details on the species used here are found in Supplementary Table S1. The selection of these species was based on their feasibility to growth in vitro and the existence of previous phenotypic evidence of some key characteristics, including adhesion to HeLa cells, biofilm formation, cytotoxic assays as well as the determination of antimicrobial tolerance [22, 23, 41, 42]. Each inoculum was grown in sBHI [Brain-heart infusion (Liofilchem, Rosetodegli Abruzzi, Italy) supplemented with 2% (wt/wt) gelatin (Liofilchem), 0.5% (wt/wt) yeast extract (Liofilchem), and 0.1% (wt/wt) soluble starch (Panreac, Barcelona, Spain)] for 24 h at 37 °C with 10% CO_2_ (Shel Lab, Cornelius, Oregon, USA) [23]. The exceptions were with consortia with the strict anaerobes *A. vaginae, M. mulieris*, and *P. bivia* [43], which were grown in sBHI and incubated at 37 °C, under strict anaerobic conditions (AnaeroGen Atmosphere Generation system; Oxoid, United Kingdom).

### Coaggregation assays

To determine the extent of the coaggregation between *G. vaginalis* and BV-associated bacteria we used an experimental model developed by Reid et al. [44]. In brief, 500 µL of *G. vaginalis* (10^7^ CFU/mL) was combined with 500 µL of each BV-associated species (10^7^ CFU/mL) in 24-well plates (Thermo Fisher Scientific, Lenexa, KS, USA). Then, bacteria were incubated for 4 h, at 37 °C, in 10% CO_2_. The aggregates were visualized using an inverted light microscope Leica DMI 3000B (Leica Microsystems GmbH, Wetzlar, Germany) and the score was evaluated as following: 0, no aggregation; 1, small aggregates comprising small visible clusters of bacteria; 2, aggregates comprising larger numbers of bacteria, settling to the center of the well; 3, macroscopically visible clumps comprising larger groups of bacteria which settle to the center of the well; 4, maximum score allocated to describe a large, macroscopically visible clump in the center of the well. Auto-aggregation was assessed for each bacterial strain. All assays were performed in duplicate and repeated in three different days.

### Biofilm formation

Dual-species biofilms were formed using an in vitro model previously developed [21], in support of the hypothesis that *G. vaginalis* is the early colonizer in BV, serving as a scaffold for other bacterial species incorporation [45]. Briefly, the cell concentration of *G. vaginalis* was assessed by optical density (OD) at 600 nm and this initial culture was further diluted in order to obtain a final concentration of approx. 10^7^ CFU/mL (OD_600_ nm = 0.15). After homogenization, 500 μL of *G. vaginalis* suspensions were dispensed into each well of a 24-well flat-bottom tissue culture plate (Orange Scientific, Braine L’Alleud, Belgium). The tissue cultured plates were then placed in an incubator at 37 °C in 10% CO_2_. Following 24 h of biofilm formation, the planktonic cells were removed carefully and 500 μL of fresh medium was added to each well. At the same time, the suspension of second BV-isolate was added (in a concentration approx. 10^7^ or 10^5^ CFU/mL; Supplementary Table S1) to each well and the plates were further incubated for 24 h. Then, the dual-species biofilms were washed once with phosphate buffer saline (PBS) solution. The 24 or 48 h mono-species biofilm of *G. vaginalis* was used as a control. sBHI was used as a negative control in all experiments to exclude any possible contamination. All assays were repeated three times with four technical replicates.

### PNA FISH hybridization and DAPI staining

To quantify the total cells of mono- and dual-species biofilms, we used the method suggested by Freitas et al. [46]. In brief, biofilms were scraped and resuspended in PBS. The total cells of the mono- or dual-species biofilms were quantified using a Neubauer chamber coupled with an Olympus BX51 epifluorescence microscope equipped with a CCD camera (DP72; Olympus, Lisboa, Portugal). Cell suspensions were stained with 4′-6-diamidino-2-phenylindole (DAPI, 2.5 μg/mL). DAPI staining was detected in a specific filter, BP 365–370, FT 400, LP 421 present in the microscope. Next, we discriminated the bacterial population of biofilm by using the peptide nucleic acid fluorescence in situ hybridization (PNA FISH) method as previously described [47]. Briefly, after fixing the biofilm suspension, a PNA probe specific for *G. vaginalis* (Gard162) was added to each well of epoxy-coated microscope glass slides (Thermo Fisher Scientific). An additional staining step was done at the end of the hybridization procedure, covering each glass slide with DAPI. Microscopic visualization was performed using filters capable of detecting the PNA probe (BP 530–550, FT 570, LP 591 sensitive to the Alexa Fluor 594 molecule attached to the Gard162 probe) and DAPI (as described above). An external control was performed to determine the sensitivity of the PNA probe for several dilutions of 48 h *G. vaginalis* mono-species biofilm cells, correlating the DAPI with PNA FISH counts.

### Confocal laser scanning microscopy analysis of biofilm bacterial distribution

To analyze the bacterial distribution of dual-species biofilms, the biofilm structure was evaluated by confocal laser scanning microscopy (CLSM) using the PNA Gard162 probe coupled to DAPI staining as we described above. For this experiment, biofilms were formed on an eight-well chamber slide (Thermo Fisher Scientific™ Nunc™ Lab-Tek™, Rochester, NY, USA) at 37 °C in 10% CO_2_ for 48 h with replacement of sBHI medium at 24 h of growth and the addition of the respective second BV-associated bacteria. The CLSM images were acquired in an Olympus™ FluoView FV1000 (Olympus) confocal scanning laser microscope, using a ×40 objective. Images were acquired with 512 × 512 resolutions at four different regions of each surface analyzed.

### Gene expression quantification

Dual-species biofilms were grown as described above. Gene expression of six potential virulence genes, namely vaginolysin (*vly*), sialidase (*sld*), *HMPREF0424_0821, HMPREF0424_1122, HMPREF0424_0156*, and *HMPREF0424_1196*, was determined according to our previous study [37]. For each tested condition, total RNA from a biofilm pooling (10 wells of a 24-well-plate) was extracted using an ExtractME RNA Bacteria & Yeast kit (Blirt S.A., Poland) with minor changes, as optimized before [48]. Next, genomic DNA was degraded with one step of DNase treatment (Fermentas, Lithuania) following the manufacturer’s instructions. RNA concentration, purity, and integrity were determined as described before [49]. The same amount of total RNA (300 ng/μL) was reverse transcribed using the RevertAid™ First Strand cDNA synthesis kit (Fermentas), as previously optimized, and gene-specific reverse transcription primers as a priming strategy. Quantitative PCR (qPCR) was prepared by mixing together 5 µL of iQ SYBR green supermix (Bio-Rad, Hercules, CA, USA), 2 µL of 1:100 diluted cDNA, 0.5 µL of 5 µM Forward and Reverse primes and water up to 10 µL. The run was performed in a CFX96^TM^ thermal cycler (Bio-Rad) with the following cycling parameters: 3 min at 95 °C, followed by 45 cycles of 10 s at 95 °C, 10 s at 60 °C, and 15 s at 72 °C. Reaction efficiency was determined by the dilution method [50]. At 60 °C all set of primers (Supplementary Table S2) used had the highest and more similar efficiencies. Furthermore, the analysis of the obtained melting curves confirmed the presence of a single peak, demonstrating the specificity of the tested primers. Normalized gene expression was determined by using the delta *C*_t_ method (*E*^Δ*C*t^), a variation of the Livak method, where Δ*C*_t_ = *C*_t_ (reference gene)−*C*_t_ (target gene) and *E* stands for the reaction efficiency experimentally determined. A non-reverse transcriptase control was included in each reaction. At least three biologic replicates of each condition were performed with three technical replicates.

### Statistical analysis

All numerical data were subjected to statistical analysis using the independent samples *t*-test, paired sample *t*-test, or non-parametric Mann–Whitney *U* test for the data that did not follow a normal distribution according Kolmogorov–Smirvon’s test, with the statistical software package SPSS 17.0 (SPSS Inc. Chicago, IL). Results are presented as mean ± standard deviation (s.d.) or as mean ± standard error of mean (s.e.m.), unless stated otherwise.

## Results

### Co-aggregation between *G. vaginalis* and other BV-associated isolates

**Fig. 1:**
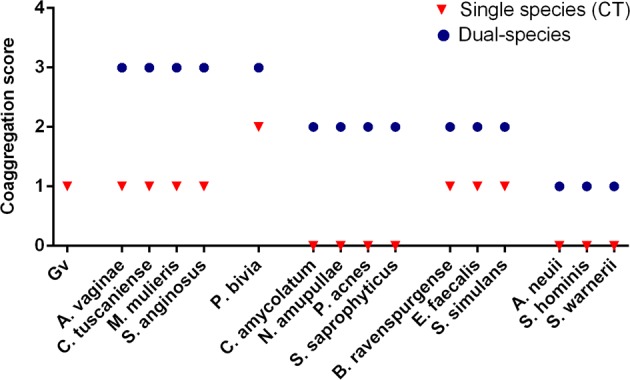
Coaggregation score of mono- or dual- bacterial species. Coaggregation score was evaluated as following: 0, no aggregation; 1, small aggregates comprising small visible clusters of bacteria; 2, aggregates comprising larger numbers of bacteria, settling to the center of the well; 3, macroscopically visible clumps comprising larger groups of bacteria which settle to the center of the well; 4, maximum score allocated to describe a large, macroscopically visible clump in the center of the well. Auto-aggregation was assessed for each bacterial species, corresponding to the experimental control (CT). Each data point represents the mode

It has been described that coaggregation is highly specific and considered a virulence factor, since microbial aggregates are a common mechanism for the survival of bacteria in nature [51, 52]. Thus, our first aim was to analyze whether BVGv and other BV-associated bacteria could co-aggregate. As shown in Fig. 1, our data demonstrated that the distinct BV-associated species co-aggregate with BVGv in different degrees. Interestingly, *A. vaginae*, *C. tuscaniense*, *M. mulieres, P. bivia*, and *S. anginosus* were the bacterial species that caused the most pronounced effect in increasing microbial aggregates in dual-species cultures.

### In vitro PNA Gard162 probe specificity

Despite the PNA Gard162 probe specificity having been previously tested for 22 *G. vaginalis* strains and for 27 other bacterial species commonly found in BV-associated microflora [47], here we also analyzed the probe specificity for the bacterial species that were not previously determined.Thus, we carried out an experiment in order to detect any possible cross-hybridization with any of the BV-associated species used herein (Supplementary Table S1). Based on these results, the Gard162 probe hybridized with a *G. vaginalis* strain, whereas no hybridization was observed for the other species tested, showing a specificity of 100% as previously reported [47].

### Quantification of bacterial populations in dual-species biofilms by PNA FISH

**Fig. 2:**
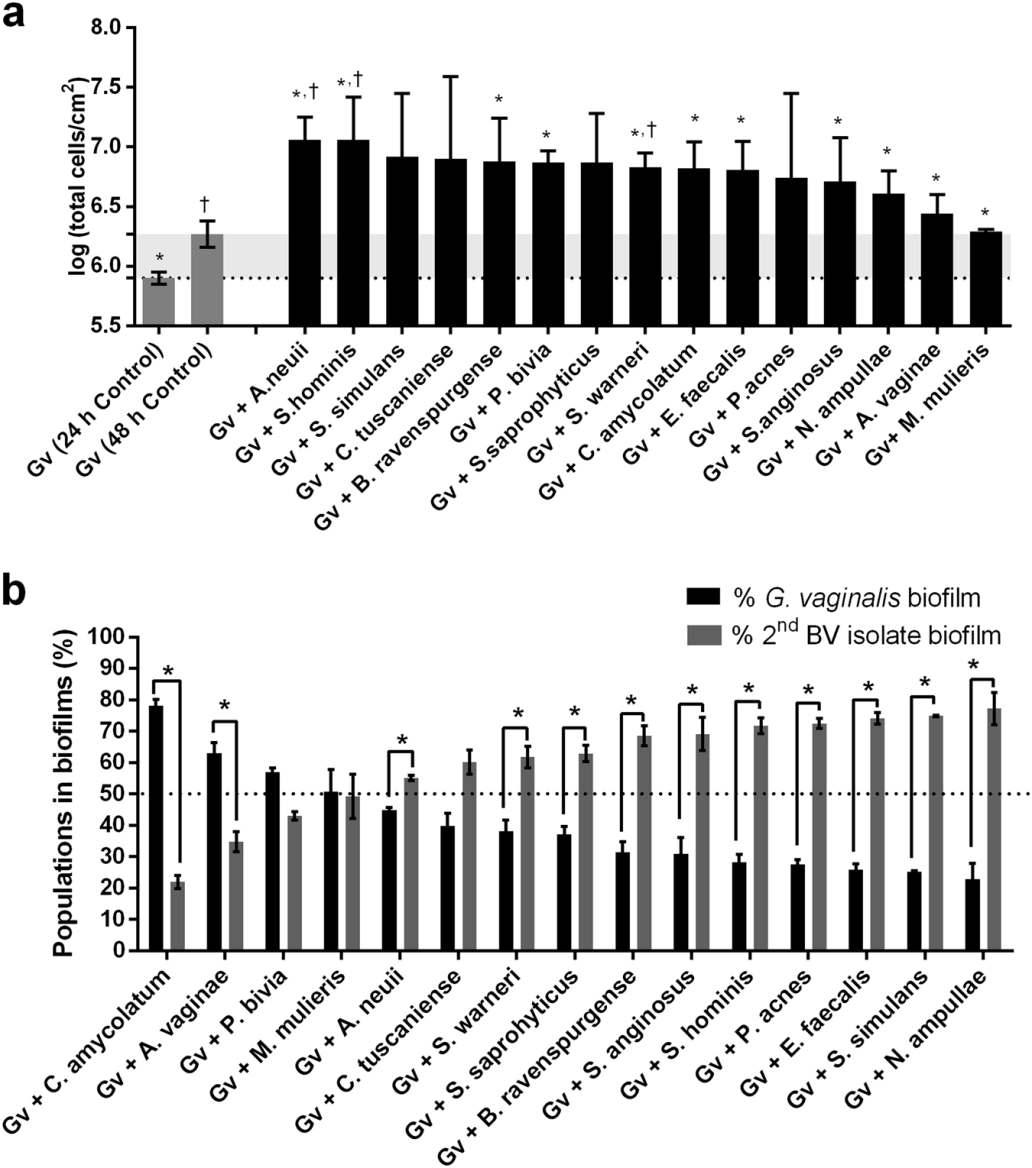
Biofilm formation profiles for each BV-associated species consortium (10^7^ CFU/mL of BVGv and 10^7^ CFU/mL of BV-associated bacteria) on dual-species biofilms. **a** Total cells counts by DAPI for mono- (*G. vaginalis* controls) and dual-species biofilms. **b** Total percentage of cells detected by PNA FISH for 48 h biofilms. Each data point represents the mean ± s.d.. *, † Values are significantly different between the dual-species consortium and the mono-species*G. vaginalis* biofilm for 24 and 48 h, respectively (independent samples *t*-test, *P* < 0.05 for **a**). * Values are significantly different between the bacterial populations of *G. vaginalis* and second BV-associated in dual-species biofilms (paired samples *t*-test for **b**, *P* < 0.05)

Taking advantage of the robustness of the PNA FISH/DAPI method (Supplementary Figure S1) for the differentiation between *G. vaginalis* and other BV-associated species, we discriminated the bacterial populations into dual-species BV-associated biofilms. Initially, we assessed the total cell number in each consortium by DAPI staining. Our results demonstrated that all tested dual-species biofilms showed a considerable enhancement of the total number of cells, as compared with mono-species BVGv biofilms (Fig. 2a). However, under our in vitro conditions, we showed that most of the dual-species biofilms had slightly higher concentrations of the second BV-associated species (Fig. 2b), in contrast to previous in vivo FISH observations [6, 29]. The only exceptions were when *A. vaginae*, *C. amycolatum*, *P. bivia*, or *M. mulieris* were added onto a pre-established BVGv biofilm.

### Analysis of dual-species biofilms by scanning and confocal microscopy

**Fig. 3:**
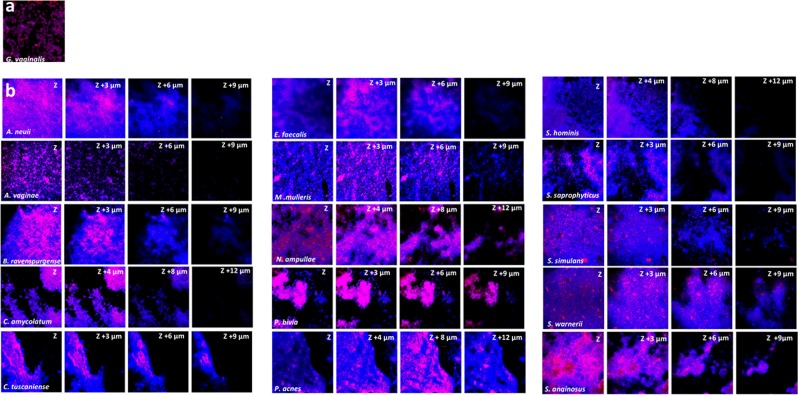
An example data set on the organization of the dual-species BV-associated biofilm for 48 h by confocal laser scanning microscopy (CLSM). **a** *G. vaginalis* mono-species biofilm labeled with PNA-probe Gard162 and DAPI staining corresponding to an experimental control. **b** CLSM images of dual-species biofilms for all 15 bacterial consortia. Images were acquired with 512 × 512 resolutions at four different regions of each surface analyzed

**Fig. 4:**
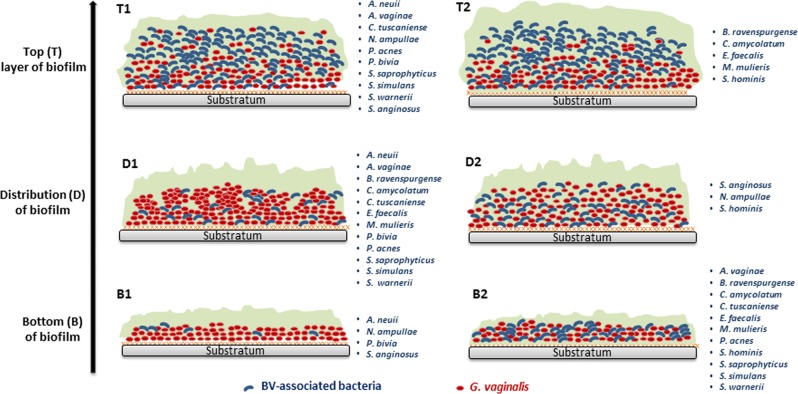
Schematic representation of the distribution of dual-species BV-associated biofilm structure from the bottom to the biofilm top. (Bottom 1—B1) Predominantly *G. vaginalis* with rare spots of second BV-isolate in the bottom; (Bottom 2—B2) both species in the bottom; (Distribution 1—D1) *G. vaginalis* exists on clusters in the biofilm; (Distribution 2—D2) *G. vaginalis* is well distributed in the biofilm; (Top 1—T1) *G. vaginalis* is reduced from the bottom to the top; (Top 2—T2) *G. vaginalis* is absence on the top layer of biofilm

The combined use of FISH with CLSM has been a useful tool to provide a better understanding of the distribution of bacterial population within the multispecies biofilms [53, 54]. Thus, in order to visualize the spatial distribution and different architectures of the tested dual-species biofilms, we analyzed different z-stacks among the 15 bacterial consortia by FISH/CLSM. As shown in Fig. 3, we were able to conclude that a second-BV species could differentially associate with a pre-established *G. vaginalis* biofilm. We grouped the bacterial consortia with an apparent similar spatial arrangement in the dual-species biofilm, using three criteria for bacterial distribution: presence in the top (T); and bottom (B); layers of the biofilm, as well as the relative distribution and aggregation within the biofilm (D). For each criterion, we found two main phenotypes, as represented in schematic Fig. 4.

Taken together, these observations indicate that only in 27% of the tested bacterial consortia, the biofilm bottom was predominantly composed of BVGv, with rare spots of second BV-associated bacteria (see B1 in Fig. 4). Otherwise, we noted that in the majority of the consortia, the secondary BV-associated species were able to incorporate the lower layers of this in vitro dual-species pre-formed BVGv biofilm (see B2 in Fig. 4). Conversely, in 33% of the consortia, BVGv was absent in the top layers of the biofilm (see T2 in Fig. 4), whereas in the remaining cases a reduced concentration was observed going from the bottom to the top of the biofilm (see T1 in Fig. 4). Interestingly, from the bottom to the top layer of the biofilm, we observed that the majority of bacterial consortia (80%) were not well distributed in a typical coaggregation structure [55, 56] (see D2 in Fig. 4), but were rather characterized by separate spatial clusters of *G. vaginalis* (see D1 in Fig. 4), leading to the incorporation of BV-associated bacteria in low numbers.

### Expression of critical genes related with *G. vaginalis* virulence can be altered in dual-species biofilms

Changes in *G. vaginalis* transcriptome during the establishment of polymicrobial BV biofilms could be a key for unraveling whether the inter-species interplay enhances the virulence of *G. vaginalis*. Thus, to decipher the impact of the second-BV species on *G. vaginalis* pathogenicity, we analyzed the expression of genes related to cytotoxicity, biofilm formation, antimicrobial resistance, and evasion of the immune system [37], in cells from mono- and dual-species biofilms.

**Fig. 5:**
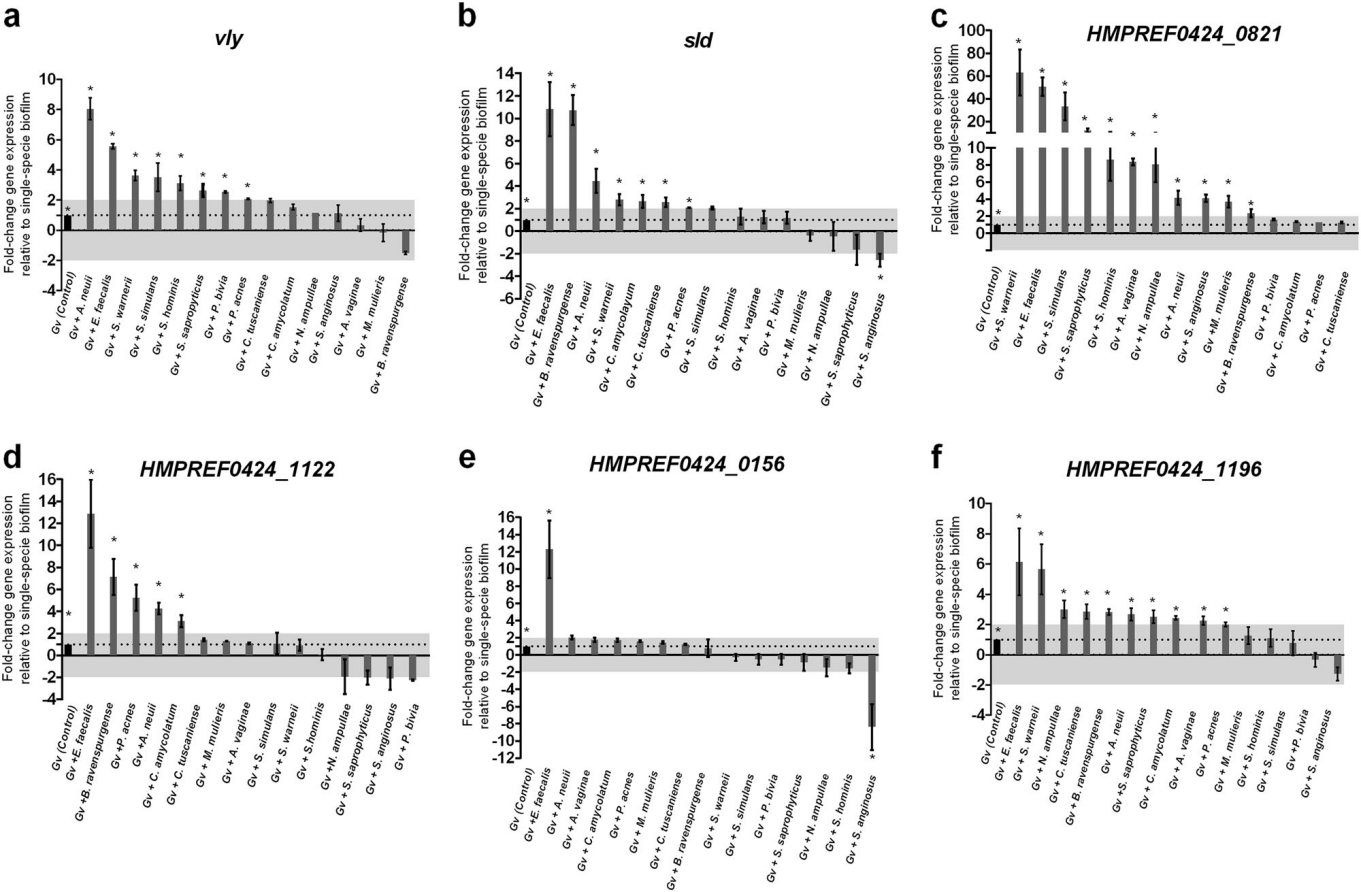
Quantification of the transcription of known virulence genes in *G. vaginalis* cultured under dual- and mono-species biofilms. **a** Quantification of vaginolysin (*vly*) transcription. **b** Quantification of sialidase (*sld*) transcription. **c** Quantification of *HMPREF0424_0821* transcript, which encodes type II glycosyl-transferase. **d** Quantification of *HMPREF0424_1122* transcript, which encodes a multidrug ABC transporter. **e** Quantification of *HMPREF0424_0156* transcript, which encodes Bacitracin transport, ATP-binding protein BcrA. **f** Quantification of *HMPREF0424_1196* transcript, which encodes a Rib-protein. The data indicate the fold-change expression of genes in *G. vaginalis* dual-species compared to mono-species *G. vaginalis* biofilm cells. For qPCR experiments, the bars represent the mean and the error bars the standard error of the mean (mean ± s.e.m.). * Values are significantly different between the dual-species consortium and the mono-species *G. vaginalis* biofilm under the same conditions (non-parametric Mann–Whitney *U*, *P* < 0.05)

*G. vaginalis* produces the toxin vaginolysin (*vly*), which might induce lysis in vaginal cells membranes [57]. Notably, our results indicated that in dual-species biofilms, the expression levels of *vly* were greatly up-regulated when *G. vaginalis* was associated with *A. neuii* or *E. faecalis* ( *P*< 0.05; Fig. 5a). Furthermore, most of the other tested species also induced a slight increase in *vly* expression, *B. ravenspurgense* being the only species that repressed *G. vaginalis vly* expression. Regarding sialidase (*sld*), which facilitates the destruction of the protective mucus layer on the vaginal epithelium [58], *E. faecalis*, *B. ravenspurgense*, or *A. neuii* considerably up-regulated its expression on BVGv. Conversely, *S. anginosus* caused a statistically significant (*P <* 0.05) reduction of *sld* expression (Fig. 5b).

It has been proposed that glycosyltransferases are likely to be important for the biosynthesis of exopolysaccharide, which in turn is important for the biofilm formation required for the full virulence of *G. vaginalis* [37, 59]. Not surprisingly, the expression of *HMPREF0424_0821* transcript, which encodes glycosyltransferases type II, was up-regulated in all consortia, with statistical significance in 73% of the tested dual-species biofilms (Fig. 5c). Nevertheless, further studies are required to clarify whether the overexpression of this transcript by BVGv could be caused by an enhancement of exopolysaccharide production or by a metabolic shift that could occur in the presence of secondary BV-associated species.

We also tested the expression of transcripts encoding antimicrobial-specific resistance proteins belonging to efflux pump families (*HMPREF0424_1122* and *HMPREF0424_0156*), since it has been proposed that dual-species biofilms may confer antibiotic tolerance and resistance to mucosal immune defences [60]. Herein, the biggest difference found on the *G. vaginalis* transcriptomic profile was caused by *E. faecalis*, in which we observed, on average, an expression of approx. 12-fold higher in the dual-species biofilms than in the mono-species biofilms (*P* < 0.05; Fig. 5d, e). Contrariwise, only *S. anginosus* promoted a significantly (*P* *<* 0.05) reduction of the of transcription levels of *HMPREF0424_0156* gene (Fig. 5e).

Finally, we analyzed the expression of *HMPREF0424_1196* transcript, which encodes a Rib-protein that belongs to the α-like protein (Alp)-family of highly repetitive surface antigens [61]. These proteins elicit protective immunity through their inter-strain size variability [59]. Importantly, it was found that *HMPREF0424_1196* transcript levels were greatly elevated (*P <* 0.05) when *E. faecalis* or *S. warneii* was co-cultured with the BVGv pre-established biofilm (Fig. 5f). It is also worthwhile noting that the remaining BV-associated bacteria incited a more slight alteration in the transcription levels of *HMPREF0424_1196* gene by BVGv biofilm cells.

## Discussion

Microbial cell–cell interactions in the vaginal flora are believed to play an integral role in the development of biofilms and, ultimately, they can also generate an array of serious gynecological and obstetric complications [3, 62, 63]. The description of a polymicrobial biofilm on the epithelial surface from BV vaginal biopsy specimens puts *G. vaginalis*, the major component of these multispecies communities, at the center of BV pathogenesis [6, 27, 29, 45, 58]. However, the effects of other species found in BV-associated microflora on biofilm formation and its impact in *G. vaginalis* pathogenicity, the presumably primary etiologic agent of BV, are still poorly known [21, 27, 33, 36]. Importantly, we have previously shown that, by themselves, some BV-associated species lack key virulent traits [23]. Therefore, herein, we hypothesized that some, but not all, BV-associated species could enhance BVGv biofilms mediated virulence. We selected 15 BV-associated species previously characterized [23, 33] and assessed their interactions with BVGv using a dual-species biofilm model.

As we have demonstrated before, most of the tested BV-secondary species were able to enhance the total biomass of pre-established *G. vaginalis* biofilms [33]. Curiously, contrary to what has been described in vivo [6, 29, 64], most of our dual-species biofilms comprised slightly less than 50% of *G. vaginalis*. Discrepancies from in vitro and in vivo biofilms have been previously reported in other infections [65] and can be attributed to several factors. First, biofilm formation by *G. vaginalis* was pre-formed in tissue culture plates rather than on vaginal epithelium, where the presence of host-derived factors (e.g. mucus production, specific receptors on the epithelial surface) can influence the biofilm development. Unfortunately, this technical limitation is not easy to overcome since, as shown before, *G. vaginalis* quickly induces cytotoxic changes and detachment of pre-adhered epithelial cultures [13, 14, 22]. Furthermore, the different optimal conditions of bacterial growth can lead to discrepant bacterial growth rates [66], and consequently directly impact the composition and possible bacterial interactions within the in vitro BV biofilms. To minimize this, we repeated the co-incubation experiments, using a lower bacterial concentration in order to mimic the vaginal microflora [67], but the overall results did not change significantly (Supplementary Figure S2).

In an effort to better understand the ecological interactions between BVGv and other BV-associated species, we also analyzed the architecture and bacterial spatial organization of in vitro BV biofilms, since this remains unclear. It has been shown before that microorganisms are not randomly organized within a multispecies biofilm, but follow a pattern that contributes to the fitness of the whole community [68, 69], for example, bacteria are organized in layers, clusters, or are well-mixed [70]. This spatial organization partially determines bacterial survival when the biofilm is exposed to toxic compounds [71]. This depends to a great extent on interactions between the species and their local micro-environments in the matrix with respect to nutrient, oxygen, and metabolite gradients [72]. To date, some studies have been shedding new light on the arrangement and spatial distribution of BV-associated biofilms through the analysis of vaginal specimens by using FISH [6, 27, 29, 31, 36, 64]. These studies have mainly focused on *G. vaginalis* and *A. vaginae*. It has been proposed that the vaginal biofilm creates a favorable environment for anaerobic bacteria, due to the presence of an oxygen gradient within the biofilm. By embedding itself within the biofilm, *A. vaginae* can take advantage of the anaerobicity, proliferates, and exists in a mutualistic relationship with *G. vaginalis*. Remarkably, our present study provides new insights into the spatial distribution of multiple dual-species biofilms, since we found striking differences in the different consortia, suggesting that the type of bacterial interaction is species-specific in the presence of a polymicrobial community. Interestingly, the most predominant dual-species biofilm phenotype was characterized by the presence of both species on the biofilm bottom, with BVGv present in clusters in the intermediate layers, with higher concentration in the lower biofilm layers. This *G. vaginalis* spatial distribution in mixed biofilms could reflect a protective mode for *G. vaginalis* maintenance in adverse conditions, such as in the presence of antimicrobial compounds [73].

**Fig. 6:**
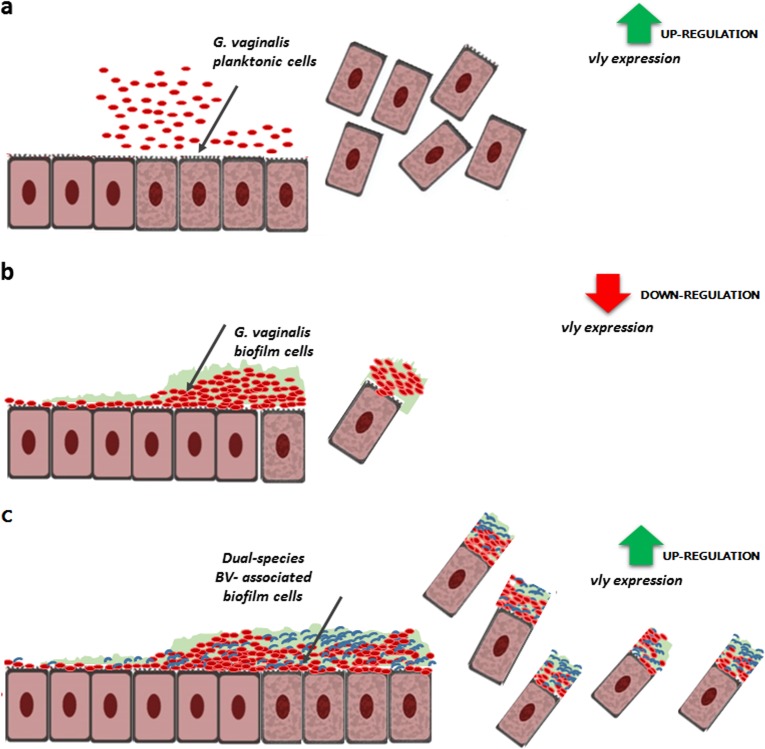
Hypothetical model of *G. vaginalis* vaginolysin (*vly*)-mediated cytotoxicity in different bacterial phenotypes **a** planktonic cells, **b** *G. vaginalis* mono-species biofilm, **c** dual-species biofilms, corresponding to a pre-formed BVGv biofilm in association with a second BV-associated bacteria

It is noteworthy that bacterial biofilms may also suppress certain virulence factors while others are activated in order to evade immune defenses and survive challenging conditions [74]. Therefore, we also analyzed how the different consortia could influence key virulence genes of *G. vaginalis* [13, 24, 37, 58, 59]. Several studies have highlighted the role of the *vly* gene in *G. vaginalis* virulence [12, 57, 75–77]. The *vly* gene belongs to the cholesterol-dependent cytolysins (CDCs), a family of pore-forming toxins, which cause cytotoxicity on vaginal epithelium [57]. Interestingly, we recently showed that *vly* expression can vary according to *G. vaginalis* phenotype, in which we found higher *vly* transcript levels in a planktonic form than in mono-BVGv biofilm cells [37]. The lower levels of expression of *vly* transcript in single biofilms might reflect the more chronic nature of vaginal colonization by BVGv and serve as a means towards preventing a host immune response. Importantly, based on our present study, we also propose that under specific ecological conditions, some BV-associated bacteria, in particular *A. neuii* or *E. faecalis*, can trigger an overexpression of *vly* transcript by BVGv cells. Consequently, the complex interplay between BVGv and specific BV-associated species can enhance vaginal desquamation and eventual formation of clue cells (Fig. 6).

The same trend was observed with *sld*, which is known to facilitate the destruction of the protective mucus layer on the vaginal epithelium by hydrolysis of sialic acid on the glycans of mucous membranes. This process possibly facilitates adhesion of bacteria on the vaginal epithelium since it has been linked with the development of biofilm [58]. Moreover, the biofilm formation was also likely to be affected by the glycosyltransferases type II [59]. Furthermore, similar to what was described in other mixed biofilm studies [60, 78], in the present study we also observed that some second BV-associated species might likely confer an increase in antibiotic tolerance and resistance to mucosal immune defenses, thereby contributing to the persistence and recurrence of BV.

Taken together, this study reveals that molecular interactions were very specific to each consortium, confirming our original hypothesis that not all BV-secondary bacteria contribute to the enhancement of BV pathogenesis by influencing *G. vaginalis* virulence. Interestingly, *E. faecalis* and *A. neuii* influenced more of the tested genes than other more commonly BV-associated species, such as *M. mulieris* or *A. vaginae* (Supplementary Figure S3). What this translates is that the mere presence of a specific bacterial species during BV does not imply that it has an active role during BV development, as previously proposed [7, 79]. While *E. faecalis* is less often found in BV, this is a known virulent species, which has also been isolated from patients with urinary tract infections [80] and vaginitis [81–83], whereas *A. neuii* can be isolated from a variety of infections [84], including genitourinary infections [85, 86]. Both bacterial species have different factors implicated in the pathogenesis [83, 87], which may contribute to aggravate the outcomes, sequelae, and recurrence of BV. In any case, more basic research is needed to fully understand the pathways and functions of these potential virulence genes [88].

Overall, the evidence from this study points towards the idea that “social networking” between co-infecting bacteria can profoundly affect the progress of BV and its clinical outcome. Therefore, more research is needed to provide a better mechanistic insight into the complex interplay between *G. vaginalis*, the very wide range of BV-associated species, and their eukaryotic hosts. Understanding the molecular basis and biological effect of these inter-bacterial processes may provide novel information necessary to define new targets and strategies for BV control.

## Supplementary information

Supplementary data
