# Supplementary material for: Unveiling the role of Gardnerella vaginalis in polymicrobial Bacterial Vaginosis biofilms: the impact of other vaginal pathogens living as neighbors
Source: ISME J. 2019 Jan 22;13(5):1306–17. doi: 10.1038/s41396-018-0337-0 (PMC6474217; doi:10.1038/s41396-018-0337-0)
Supplement: Supplementary file 1 — Supplementary data [file 41396_2018_337_MOESM1_ESM.pdf]

Supplementary data

The ISME Journal

Joana Castro[1,2] , Daniela Machado[1], Nuno Cerca[1]

[1] Centre of Biological Engineering (CEB), Laboratory of Research in Biofilms Rosário Oliveira (LIBRO), University of Minho, Campus de Gualtar, 4710-057 Braga, Portugal.
[2] Instituto de Ciências Biomédicas Abel Salazar (ICBAS), University of Porto, Rua de Rua de Jorge Viterbo Ferreira 228, 4050-313 Porto, Portugal.

* Corresponding author: nunocerca@ceb.uminho.pt

Centre of Biological Engineering (CEB), Laboratory of Research in Biofilms Rosário Oliveira (LIBRO), University of Minho, Campus de Gualtar, 4710-057 Braga, Portugal
Tel.: +351 253604423, Fax: +351 253604429.

Supplementary Table S1. Bacterial species used in PNA-FISH assays and their specificity with PNA Gard162 probe

**Bacteria (*n* = 16)**	**Accession numbers [a]**	**OD/CFU Calibration**	**Gard162 Probe efficiency [b]**	**Reference**
Actinomyces neuii UM067An	KT805271.1	$OD_{600nm} = 2\times10^{-12}$(CFU/mL) + 0.2806	-	This study
Atopobium vaginae FA	absence [c]	$OD_{600nm} = 9\times10^{-13}$(CFU/mL) + 0.0818	-	Machado et al, 2013
Brevibacterium ravenspurgense UM066Br	KT805269.1	$OD_{600nm} = 1\times10^{-9}$(CFU/mL) – 0.0585	-	This study
Corynebacterium amycolatum UM065Ca	KT805275.1	$OD_{600nm} = 6\times10^{-10}$(CFU/mL) + 0.0320	-	This study
Corynebacterium tuscaniense UM137Ct	KT805278.1	$OD_{600nm} = 7\times10^{-11}$(CFU/mL) + 0.1549	-	This study
Enterococcus faecalis UM035	KT614045.1	$OD_{600nm} = 3 \times10^{-11}$(CFU/mL) + 0.0002	-	This study
Gardnerella vaginalis UM241	KP996683.1	$OD_{600nm} = 5\times10^{-9}$(CFU/mL) + 0.1037	++++	This study
Mobiluncus mulieris ATCC 35239	NZ_GL405260.1	$OD_{600nm} = 4\times10^{-9}$(CFU/mL) + 0.010	-	Machado et al, 2013
Nosocomiicoccus ampullae UM121Na	KT805272.1	$OD_{600nm} = 3\times10^{-12}$(CFU/mL) + 0.1279	-	This study
Prevotella bivia ATCC 29303	L16475.1	$OD_{600nm} = 6\times10^{-8}$(CFU/mL) + 0.0214	-	Machado et al, 2013
Propionibacterium acnes UM034Pa	KT805265.1	$OD_{600nm} = 1\times10^{-10}$(CFU/mL) + 0.0140	-	This study
Staphylococcus hominis UM224Sh	KT923487.1	$OD_{600nm} = 3\times10^{-10}$(CFU/mL) + 0.1401	-	This study
Staphylococcus saprohyticus UM121Ss	KT923484.1	$OD_{600nm} = 4\times10^{-9}$ (CFU/mL) + 0.0238	-	This study
Staphylococcus simulans UM059Ss	KT805267.1	$OD_{600nm} = 5\times10^{-12}$(CFU/mL) + 0.6540	-	This study
Staphylococcus warnerii UM224Sw	KT923488.1	$OD_{600nm} = 3\times10^{-9}$(CFU/mL) + 0.0038	-	This study
Streptococcus anginosus UM241b	KT805274.1	$OD_{600nm} = 4\times10^{-10}$(CFU/mL) + 0.4236	-	This study

[a] The accession numbers of partial *16S ribosomal RNA* or *rpoB* gene sequence of vaginal isolates are downloadable from NCBI. [b] The PNA Probe (Gar162) efficiency was tested for each strain, with the following hybridization PNA FISH qualitative evaluation: (−) Absence of hybridization; (++) Moderate hybridization; (+++) Good hybridization; (++++) Optimal hybridization. The table shows the median value from the ten fields of view of each strain. [C] Strain isolated from a woman with BV based on Amsel criteria at Virginia Commonwealth University (VCU) Women's Health Clinic and it was kindly offered by Dr. Kimberly Jefferson.

Supplementary Table S2. Primers used in qPCR experiments

**Target gene**	**Gene description**	**Primer sequence (5' to 3')**	**T $_{melting}$ (ºC)**	**Efficiency [a] (%)**	**Amplicon size (bp)**
16S RNA	16S ribosomal RNA of *G. vaginalis*	Fw TGAGTAATGCGTGACCAACC	55.2	100	167
		Rv AGCCTAGGTGGGCCATTACC	59.3		
HMPREF0424_0103 (*vly*)	Thiol-activated cytolysin vaginolysin	Fw GAACAGCTGGGCTAGAGGTG	60.01	100	153
		Rv AATTCCATCGCATTCTCCAG	60.04		
Sld	Sialidase	Fw CCGAATTTGCGATTTCTTCT	54.00	86	189
		Rv CGTACGGAAGTTTTGGAAGC	58.00		
HMPREF0424_0821	Glycosyltransferase, group 2 family protein	Fw CAACGAAGGCATAGGTTTCC	59.57	99	156
		Rv GCGCTTGGAACTGCTTTAAC	60.02		
HMPREF0424_1122	Multidrug resistance ABC transporter	Fw CAGCACCTGTAGCTCCAACA	60.05	89	195
		Rv TGGCTCAAGAGATTGTGTGC	59.99		
HMPREF0424_0156	Bacitracin transport ATP-binding protein BcrA	Fw CCGACCGCATACCTATTTTG	60.34	90	178
		Rv GCAAGACGGTCTCCAAACTC	59.85		
HMPREF0424_1196	LPXTG-motif cell wall anchor domain-containing protein	Fw TGCAAAGACAGGCGATAGTG	60.00	99	173
		Rv TAATCGTTGCGGTTGTTTCA	60.11		

[a] PCR amplification efficiency (E) for each gene was determined from the slope of a standard curve ($E = 10^{[-1/slope]}$), generated with a 10-fold dilution series of cDNA.

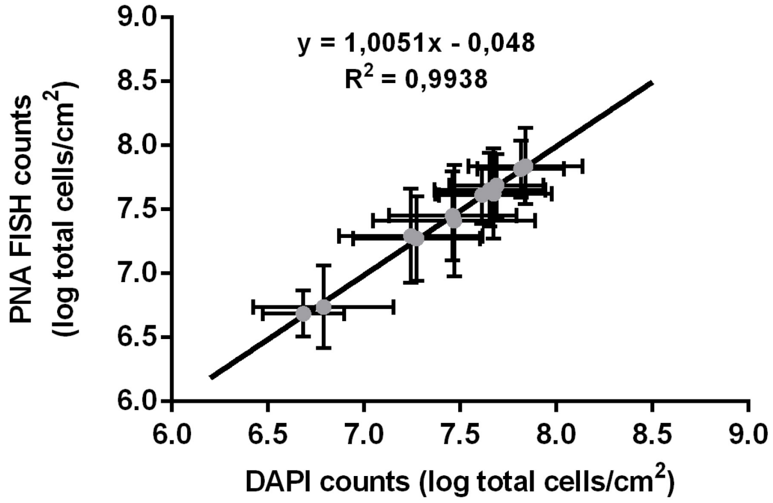

Supplementary Figure S1. Correlation between the PNA FISH counts and the DAPI counts for *G. vaginalis* at different bacterial concentrations, in which BVGv biofilm cells that were identified indirectly by DAPI coincided with the populations quantified by PNA FISH. Each data point represents the mean ± s.d..

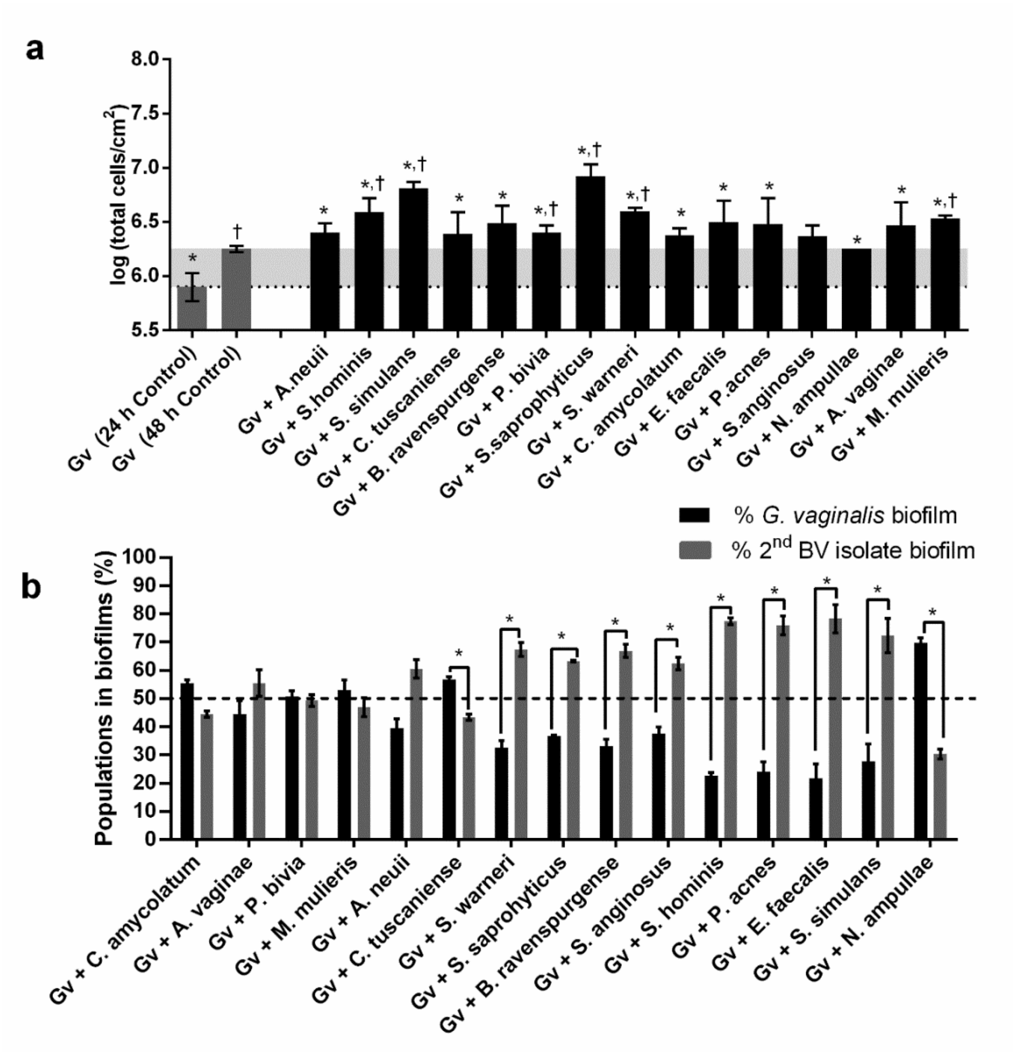

Supplementary Figure S2. Biofilm formation profiles for each BV-associated species consortium (10^7 CFU/mL of Gv & 10^5 CFU/mL of BV-associated bacteria) on dual-species biofilms. (a) Total cells counts by DAPI for mono- (*G. vaginalis* controls) and dual-species biofilms. (b) Total percentage of cells detected by PNA FISH for 48h biofilms. Each data point represents the mean ± s.d.. *, † Values are significantly different between the dual-species consortium and the single- *G. vaginalis* biofilm for 24 h and 48 h, respectively (independent samples t-test, $P < 0.05$ for figure 2a). * Values are significantly different between the bacterial populations of *G. vaginalis* and second BV-associated in dual-species biofilms (paired samples t-test for figure 2b, $P < 0.05$).

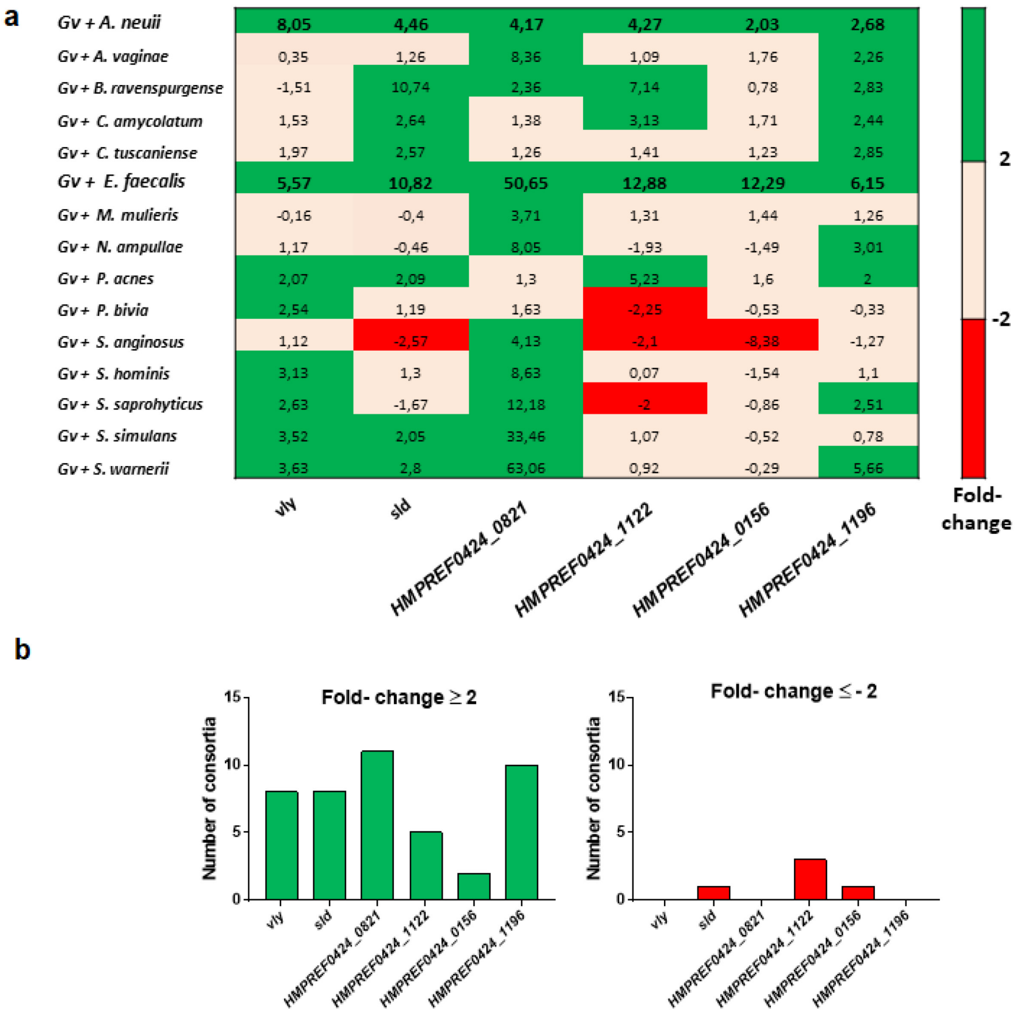

Supplementary Figure S3. Summarizing the transcript levels of all six potential virulence genes of *G. vaginalis* among 15 bacterial consortia. (a) *G. vaginalis* up-regulation in dual-species biofilms (fold-change difference ≥ 2), in which *A. neuii* and *E. faecalis* caused an overexpression of all 6 transcripts in *G. vaginalis*. Specific molecular interactions of some consortia were found, leading to a unique change in *G. vaginalis* transcriptome for *vly* (BVGv/*P. bivia* or BVGv/*S. saprophyticus* dual-species biofilms) and *HMPREF0424_0821* transcripts (BVGv/*M. mulieris* or BVGv/ *S. anginosus* dual-species biofilms). A specific down-regulation of *HMPREF0424_1122* transcript was found to BVGv/*S. saprophyticus* or BVGv/*P. bivia* dual-species biofilms. (b) Number of bacterial consortia that causes an up- or down-regulation in the six virulence genes of *G. vaginalis*.
